# Assessment of hormonal activity in patients with premature ejaculation

**DOI:** 10.1590/S1677-5538.IBJU.2016.0064

**Published:** 2017

**Authors:** Lütfi Canat, Akif Erbin, Masum Canat, Mehmet Dinek, Turhan Çaşkurlu

**Affiliations:** 1 Department of Urology, Okmeydani Training and Research Hospital, Istanbul, Turkey;; 2 Department of Urology, Haseki Training and Research Hospital, Istanbul, Turkey;; 3 Department of Endocrinology, Bayburt State Hospital, Bayburt, Turkey;; 4 Department of Urology, Kastamonu State Hospital, Kastamonu, Turkey;; 5 Department of Urology, Istanbul Medeniyet University Göztepe Training and Research Hospital, Istanbul, Turkey

**Keywords:** Hormones, Erectile Dysfunction, Premature Ejaculation

## Abstract

**Purpose:**

Premature ejaculation is considered the most common type of male sexual dysfunction. Hormonal controls of ejaculation have not been exactly elucidated. The aim of our study is to investigate the role of hormonal factors in patients with premature ejaculation.

**Materials and Methods:**

Sixty-three participants who consulted our outpatient clinics with complaints of premature ejaculation and 39 healthy men as a control group selected from volunteers were included in the study. A total of 102 sexual active men aged between 21 and 76 years were included. Premature ejaculation diagnostic tool questionnaires were used to assessment of premature ejaculation. Serum levels of follicle stimulating hormone, luteinizing hormone, prolactin, total and free testosterone, thyroid-stimulating hormone, free triiodothyronine and thyroxine were measured.

**Results:**

Thyroid-stimulating hormone, luteinizing hormone, and prolactin levels were significantly lower in men with premature ejaculation according to premature ejaculation diagnostic tool (p=0.017, 0.007 and 0.007, respectively). Luteinizing hormone level (OR, 1.293; p=0.014) was found to be an independent risk factor for premature ejaculation.

**Conclusions:**

Luteinizing hormone, prolactin, and thyroid-stimulating hormone levels are associated with premature ejaculation which was diagnosed by premature ejaculation diagnostic tool questionnaires. The relationship between these findings have to be determined by more extensive studies.

## INTRODUCTION

Premature ejaculation (PE) is the most frequent male sexual disorder. According to different criteria, its prevalence might widely vary, ranging from 8 to 30% (1) up to 22-38% (2). Over the past decade, increasingly assertive efforts have been assumed to explain the etiology of PE. Genetic, neurobiological and somatic etiologies for PE have been hypothesized: disruptions in central serotonergic neurotransmission (3), potent cortical representation of the pudental nerve (4), prostatitis (5), hypersensitivity of the penis (6), recreational drugs (7), and hormonal disorders (8-11). Although it is well described that male sexuality and reproduction are hormonally regulated, the association between endocrine control and ejaculatory reflex is still not completely elucidated. It is well reported that sex steroids play a role in the regulation of ejaculation (8) and demonstrated similar effects of thyrotropin and prolactin, but in the against direction (12). Current guidelines on PE supply little information on this circumstance (13, 14).

Stopwatch measures of IELT are commonly used in clinical trials of PE. However, they have not been recommended for use in routine diagnosis of PE (15). The International Society for Sexual Medicine (ISSM) described three common structures used in the most definitions of PE: (1) a short ejaculatory latency time (prior to or within about 1 minute of vaginal penetration (lifelong PE) or a clinically significant and bothersome reduction in latency time, often to about 3 minutes or less (acquired PE); (2) the inability to delay ejaculation on all or nearly all vaginal penetrations; (3) distress and/or avoidance of sexual intimacy (16). The requirement to evaluate PE objectively has led to the development of some questionnaires based on the use of patient-reported outcome. The premature ejaculation diagnostic tool (PEDT) was developed and validated by Symonds et al. to standardize the diagnosis of PE in clinical trials (17). In the current study, we used PEDT questionnaires to assesssment of the relationship between PE and hormonal functions.

## MATERIALS AND METHODS

The present study involved an observational and prospective design. It was conducted on a series of 102 sexual active men aged between 21 and 76 years between May 2014 and June 2015. Sixty three participants who consulted our outpatient clinics with complaints of PE and 39 healthy men as a control group selected from volunteers enlisted through an announcement about the research project were included in the study. We included patients with monogamous and heterosexual relationship with the same partner for at least 6 months.

PE was evaluated by using the Turkish version of the PEDT questionnaires which provides scores for five subdomains: control, frequency, minimal stimulation, distress, and interpersonal difficulty. Each question is scored 0-4 and higher scores indicate higher severity. Total PEDT score is calculated as the sum of the scores of the five subdomains. A PEDT score 42.6±11.8 11 indicates PE (17, 18).

Patient’s age, body mass index (BMI), smoking history, alcohol consumption and the International Index of Erectile Function Erectile Domain (IIEF-ED) score were recorded. Blood samples to evaluate the routine laboratory tests and serum levels of follicle stimulating hormone (FSH), luteinizing hormone (LH), prolactin, total testosterone, free testosterone, thyroid-stimulating hormone (TSH), free triiodothyronine (T3), free thyroxine (T4) were measured using immunoassay method, except free testosterone measurement that was performed by the enzime-linked immunosorbent assay method in all participants. Blood samples were obtained in the morning after an overnight fasting. For abnormal laboratory findings, a second blood sample was taken in a few days.

We excluded men with moderate or severe erectile dysfunction, any sexual dysfunction of partner, a sexual intercourse frequency less than once a week, any medication history due to PE, any history of neuropathic or psychological disease, hypogonadism, and concomitant chronic diseases. Participants who had used drugs that might disturb hormonal values, and who had been diagnosed with prostatitis were also excluded from the study.

Committee of Ethics approved the protocol and all participants provided written informed consent. Collected data were analyzed with Statistical Package for Social Sciences (SPSS) version 17. Statistical analysis were performed using Student t test, Mann-Whitney U test and Chi-square test. P values lower than 0.05 were considered as significant.

## RESULTS

The baseline characteristics of the 102 participants are described in Table-1. The mean ages of the participants evaluated in the PE group (n=63) and control group (n=39) were 42.6±11.8 years (23-76) and 40.3±10.7 (21-65), respectively (p=0.065). There was no significant difference between the BMI, smoking status, alcohol consumption, glucose, creatinine and lipid profiles between the two groups (p>0.05).

**Table 1:** Characteristics and hormonal status of the studied participants.

	PEDT score ≥11 (PE group)	PEDT score <11 (Control group)	p*
	n=63	n=39	
Age	42.6±11.8	40.3±10.7	0.065
BMI (kg/m^2^)	26.6±4.2	27.7±5.0	0.333
Smoking history (n, %)	32, 50.8%	20, 51%	0.962
Alcohol consumption (n/%)	8/12.7%	3/7.69%	0.428
IIEF–ED score	25.2±3.7	25.7±3.5	0.491
Glucose	113.4±43.4	95.0±8.0	0.162
Creatinine	0.8±0.1	0.9±0.1	0.186
Total cholesterol	180.2±43.4	182.7±42.3	0.635
LDL cholesterol	112.8±31.1	118.2±36.7	0.517
HDL cholesterol	41.2±9.3	43.5±10.0	0.219
Triglycerides	169.9±157.8	154.5±63.4	0.474
Free T3 (pg/dL)	3.5±0.6	3.6±0.4	0.067
Free T4 (ng/dL)	1.3±0.2	1.3±0.1	0.251
TSH (mIU/L)	1.7±1.4	2.1±1.2	0.017
FSH (mIU/mL)	7.6±7.1	5.5±3.9	0.054
LH (mIU/mL)	6.5±2.8	5.2±2.0	0.007
Prolactine (ng/mL)	9.5±6.1	11.6±5.0	0.007
Total Testosterone (ng/dL)	4.4±1.7	4.5±1.5	0.598
Free Testosterone (pg/dL)	12.3±4.0	12.2±3.3	0.920

* **T** test/Mann-whitney U test/Chi-square test. **PEDT** = Premature Ejaculation Diagnostic Tool; **BMI** = Body mass index; **IIEF-ED** = International Index of Erectile Function-Erectile Domain; **LDL** = Low Density Lipoprotein*;* **HDL** = High Density Lipoprotein; **TSH** *=* Thyroid-stimulating hormone; **FSH** = Follicle-stimulating hormone; **LH** = Luteinizing hormone.

The mean PEDT score in patients with PE was found to be 15.31 and in the control group, the mean PEDT score was 5.43. Of the participants, 63 scored 11 and higher (61.7%), 39 scored lower than 11 (38.2%).

The mean IIEF-ED scores did not differ between the groups. Based on IIEF-ED evaluation, the mean scores of the subjects in PE group was 25.2±3.7, while in the control group was 25.7±3.5 (p=0.491).

There was no significant difference between the levels of FSH, total testosterone, free testosterone, free T3 and free T4 between the two groups, whereas lower levels of TSH, LH and prolactin were reported in the PE group (p=0.017, 0.007 and 0.007, respectively). In the PE group mean serum TSH, LH and prolactin levels were 1.7±1.4mIU/mL, 6.5±2.8mIU/mL and 9.5±6.1ng/mL, respectively. LH level (OR, 1.293; p=0.014) was found to be an independent risk factor for premature ejaculation Table-2.

**Table 2:** Multivariate model for PEDT score and LH level.

PEDT		
	Reference	p^*^
LH	1.293 (1.054-1.585)	0.014

* Spearman correlation. **PEDT =** Premature Ejaculation Diagnostic Tool; **LH =** Luteinizing hormone.

## DISCUSSION

Although PE is a common type of male sexual disorder, it is poorly understood and patients may be misdiagnosed and mistreated (19). The pathophysiology of PE is largely unknown and hormones might have a potential role in the pathophysiology of PE. The main aim of our study was to investigate the role of several hormones in the etiology of PE.

Corona et al. reported for the first time that several testosterone levels might lead to different severities of ejaculatory disorders. They reported higher testosterone levels in their youngest subjects suffering from PE while the elder group with delayed ejaculation had lower testosterone levels (20). In our study, nevertheless, total testosterone and free testosterone levels were not significantly different in the two groups. A reasonable clarification for this is related to our inadequate sample size. Therefore, androgen suppression is not an acceptable treatment for PE yet.

In the current study, lower levels of prolactin were associated with PE; however, low prolactin levels are not a risk factor for PE. The effect of high prolactin levels has been extensively researched, but the effects of low prolactin levels have not received similar interest. In the European Male Ageing Study (EMAS) showed that low prolactin levels were associated with lower ejaculatory time on almost 3000 subjects (21). Low prolactin levels can be the outcomes of an increased dopamine activity. The use of dopamine antagonists (haloperidole etc.) induces a dramatic increases ejaculatory latency time in a dose dependent manner (22). Therefore, an increased dopaminergic tone (reflected by low prolactin levels) can explain the association with PE. Otherwise, men with lower levels of prolactin showed the highest level of free-floating anxiety and related to psychobiological features such as anxiety symptoms and PE (23).

The role of thyroid hormones in PE has been assumed. Associations between premature and delayed ejaculation with hyperthyroidism and hypothyroidism, respectively, have been recorded in animal models and humans (24-26). Treatment of hyperthyroidism has verified to be effective for PE; the prevalence of PE fell from 50 to 15%, after normalizing thyroid function in men with hyperthyroidism (24). In our study, lower levels of TSH were reported in the participants with PE than control group. We suggest that laboratory confirmation should be advised in men with PE inasmuch as hyperthyroidism increased the risk of all-cause mortality (27) .

Mohseni et al. found no correlation between LH level and PE, even though men with PE had significantly higher levels of FSH (9). However, in our study there was no correlation between PE and FSH level, but men with PE had lower levels of LH than control group. Moreover, we found that LH is an independent risk factor for PE. The literature evidence regarding the gonadotropins related to men with PE is still lacking.

In the present study, we used the PEDT score which was developed and validated by Symonds et al. for diagnosis of PE (17). Rowland and Kolba advocated that clinicians should approach the 1 minute IELT criterion with flexibility, considering IELTs up to 2 minutes for a PE. Finally, PE cannot be diagnosed simply by the IELT alone (28). Kam et al. and Pakpour et al. demonstrated that the PEDT was highly effective in detecting the presence of PE and the result of their study supports its validity as a diagnostic tool in the clinical setting (29, 30). To the best of our knowledge, this is the first study to investigate the association of hormones and PE in which was used PEDT questionnaires.

Relatively small sample size is the major limitation of our study. Furthermore, patients with delayed ejaculation were not included in the study. It is obvious that there is a need for further large-scale studies which include other hormones such as oxytocin and adrenal steroids.

## CONCLUSIONS

We showed an association between hormonal activity and PE. Patients with PE have lower LH, TSH, and prolactin levels compared with normal men and the lower levels of LH is an independent risk factor for PE. The results of this study showed that the role of endocrinologic factors should be more investigate in the etiology of PE. We think that hormones might also have a potential role in the pathophysiology of PE. Perhaps hormonal therapy of PE will ameliorate the sexual and the general health of these patients. Furthermore, we think that laboratory confirmation for hyperthyroidism should advise in men with ejaculatory disorders.
